# Supplementary material for: Knockout of liver fluke granulin, Ov-grn-1, impedes malignant transformation during chronic infection with Opisthorchis viverrini
Source: PLoS Pathog. 2022 Sep 22;18(9):e1010839. doi: 10.1371/journal.ppat.1010839 (PMC9531791; doi:10.1371/journal.ppat.1010839)
Supplement: S1 Fig — Each group of flukes was subjected to gene editing targeting Ov-grn-1 (ΔOv-grn-1 flukes), Ov-tsp-2 (ΔOv-tsp-2 flukes), or with an irrelevant guide RNA as a control (Control). Relative transcript levels were plotted for both the Ov-grn-1 (A) and Ov-tsp-2 genes (B) for all three groups: control, ΔOv-grn-1, and ΔOv-tsp-2 flukes. Each panel shows ddCt (delta-delta cycle threshold) biological replicate values plotted relative to newly excysted juvenile (NEJ) control average. Resampling with replacement bootstrap analysis (B = 1000) of ddCT scores used to generate population average denoted by thick colored line and 95% confidence interval bars. (DOCX) [file ppat.1010839.s001.docx]

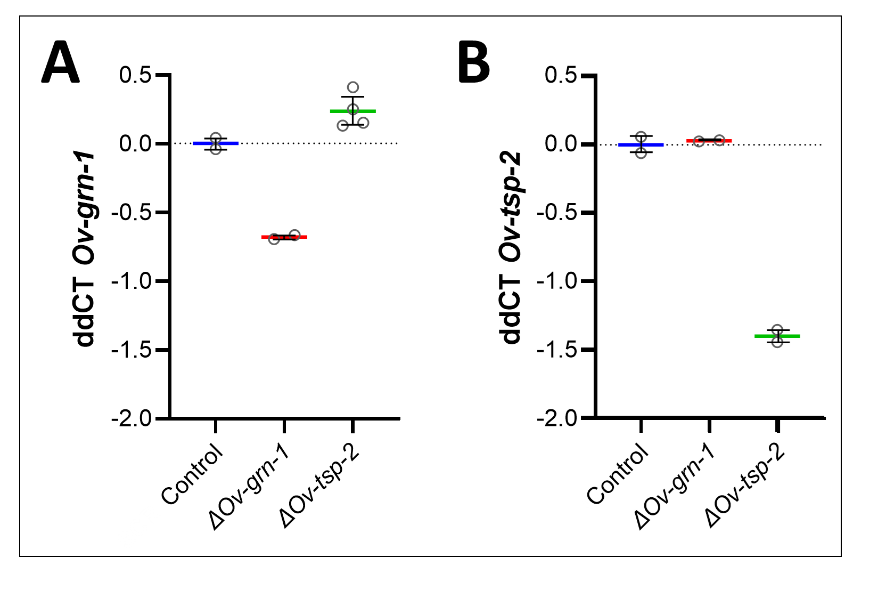


**S1 Fig. Transcript levels of gene edited NEJ flukes with bootstrapped population values**. Each group of flukes was subjected to gene editing targeting *Ov-grn-1* (*ΔOv-grn-1* flukes), *Ov-tsp-2* (*ΔOv-tsp-2* flukes), or with an irrelevant guide RNA as a control (Control). Relative transcript levels were plotted for both the *Ov-grn-1* **(A)** and *Ov-tsp-2* genes **(B)** for all three groups: control, *ΔOv-grn-1*, and *ΔOv-tsp-2* flukes. Each panel shows ddCt (delta-delta cycle threshold) biological replicate values plotted relative to newly excysted juvenile (NEJ) control average. Resampling with replacement bootstrap analysis (B = 1000) of ddCT scores used to generate population average denoted by thick colored line and 95% confidence interval bars.
