# Supplementary material for: Knockout of liver fluke granulin, Ov-grn-1, impedes malignant transformation during chronic infection with Opisthorchis viverrini
Source: PLoS Pathog. 2022 Sep 22;18(9):e1010839. doi: 10.1371/journal.ppat.1010839 (PMC9531791; doi:10.1371/journal.ppat.1010839)
Supplement: S2 Fig — Assessment of eggs per gram of feces (EPG) at 10 (A) and 12 (B) weeks after infection compared to worm burden at necropsy (week 14). The worm burden for each hamster was plotted against the EPG at weeks 10 and 12. Comparing these timepoints (C) in a linear regression analysis did not reveal variation from a line with zero slope (horizontal line) at either interval. Each hamster was designated by letter, C = control, G = ΔOv-grn-1, T = ΔOv-tsp-2, with the number of the hamster, as 1, 2, and 3. (DOCX) [file ppat.1010839.s002.docx]

**
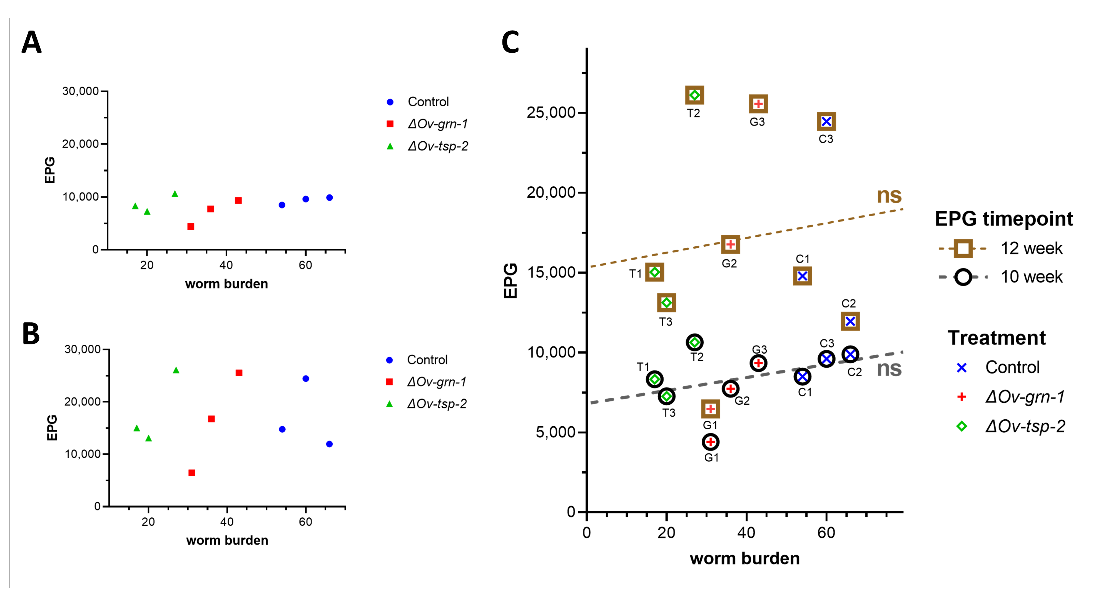
**

**S2 Fig. Experiment 1: Relationship of fecal egg count to worm burden**. Assessment of eggs per gram of feces (EPG) at 10 **(A)** and 12 **(B)** weeks after infection compared to worm burden at necropsy (week 14). The worm burden for each hamster was plotted against the EPG at weeks 10 and 12. Comparing these timepoints (**C)** in a linear regression analysis did not reveal variation from a line with zero slope (horizontal line) at either interval. Each hamster was designated by letter, C = control, G = *∆Ov-grn-1*, T = *∆Ov-tsp-2*, with the number of the hamster, as 1, 2, and 3.
