# Supplementary material for: Knockout of liver fluke granulin, Ov-grn-1, impedes malignant transformation during chronic infection with Opisthorchis viverrini
Source: PLoS Pathog. 2022 Sep 22;18(9):e1010839. doi: 10.1371/journal.ppat.1010839 (PMC9531791; doi:10.1371/journal.ppat.1010839)
Supplement: S4 Fig — Each group of the 24-wk-old flukes was subjected to gene editing targeting Ov-grn-1 (ΔOv-grn-1 flukes), Ov-tsp-2 (ΔOv-tsp-2 flukes), or with an irrelevant guide RNA as a control (Control). Each panel shows ddCt (delta-delta cycle threshold) for individual flukes plotted relative to wild-type (WT) fluke transcript levels for both Ov-grn-1 (A) and Ov-tsp-2 genes (B). The dashed line purple box inset is an enlarged section of panel B for clarity. Resampling with replacement bootstrap analysis (B = 1000) of ddCT scores used to generate population average denoted by thick colored line and 95% confidence interval bars. (DOCX) [file ppat.1010839.s004.docx]

*
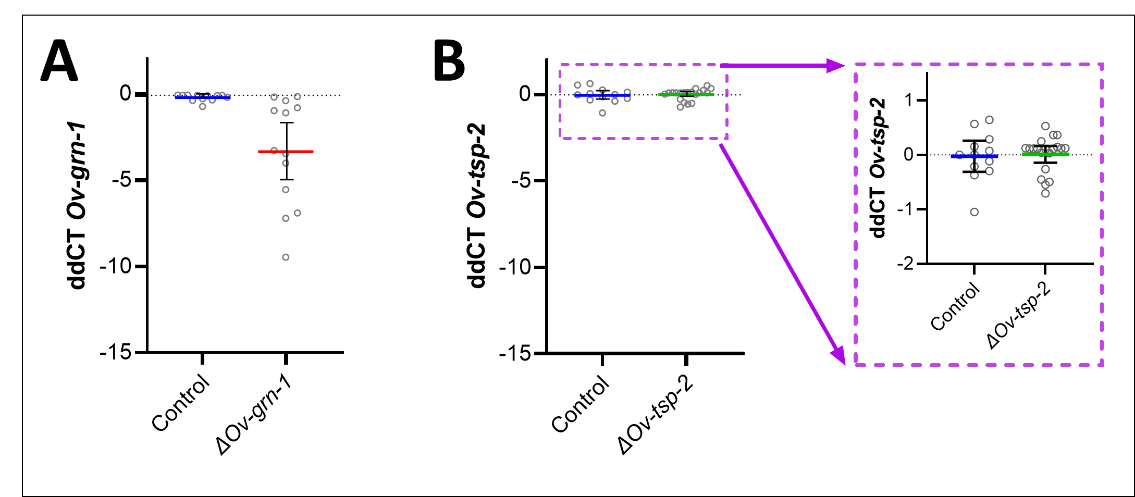
*

**S4 Fig. Transcript levels of adult flukes from Experiment 2 with bootstrapped population values**. Each group of the 24-wk-old flukes was subjected to gene editing targeting *Ov-grn-1* (*ΔOv-grn-1* flukes), Ov-tsp-2 (*ΔOv-tsp-2* flukes), or with an irrelevant guide RNA as a control (Control). Each panel shows ddCt (delta-delta cycle threshold) for individual flukes plotted relative to wild-type (WT) fluke transcript levels for *both Ov-grn-1* (A) and *Ov-tsp-2* genes (B). The dashed line purple box inset is an enlarged section of panel B for clarity. Resampling with replacement bootstrap analysis (B=1000) of ddCT scores used to generate population average denoted by thick colored line and 95% confidence interval bars.
