# Supplementary material for: Knockout of liver fluke granulin, Ov-grn-1, impedes malignant transformation during chronic infection with Opisthorchis viverrini
Source: PLoS Pathog. 2022 Sep 22;18(9):e1010839. doi: 10.1371/journal.ppat.1010839 (PMC9531791; doi:10.1371/journal.ppat.1010839)
Supplement: S5 Fig — Nucleotide substitution profiles detected in the 173 bp amplicon spanning the programmed cleavage site in Ov-grn-1 from both juvenile (NEJ) and single adult O. viverrini flukes of the ΔOv-grn-1 treatment group compared with the irrelevant guide RNA-treated control group. RGEN’s CRISPR-sub analysis tool, http://www.rgenome.net/crispr-sub/#!, aligns read-pairs to plot the substitution patterns among Illumina sequence reads from amplicon libraries from CRISPR/Cas9 editing-focused datasets. Experimental group (red, upper axis) versus control group (blue, lower axis); the X-axis shows the targeted gene including programmed cleavage site (position 0) between nucleotides 19 and 20 of ORF 1 of Ov-grn-1. Juvenile flukes are shown in the top left and each adult fluke is shown separately and designated with the number of its host hamster number (x) 1–15 and worm number (y) 1–3: (h”x” worm”y”). Substantial differences were not apparent in patterns of substitutions detected among the experimental and control groups. (DOCX) [file ppat.1010839.s005.docx]

***
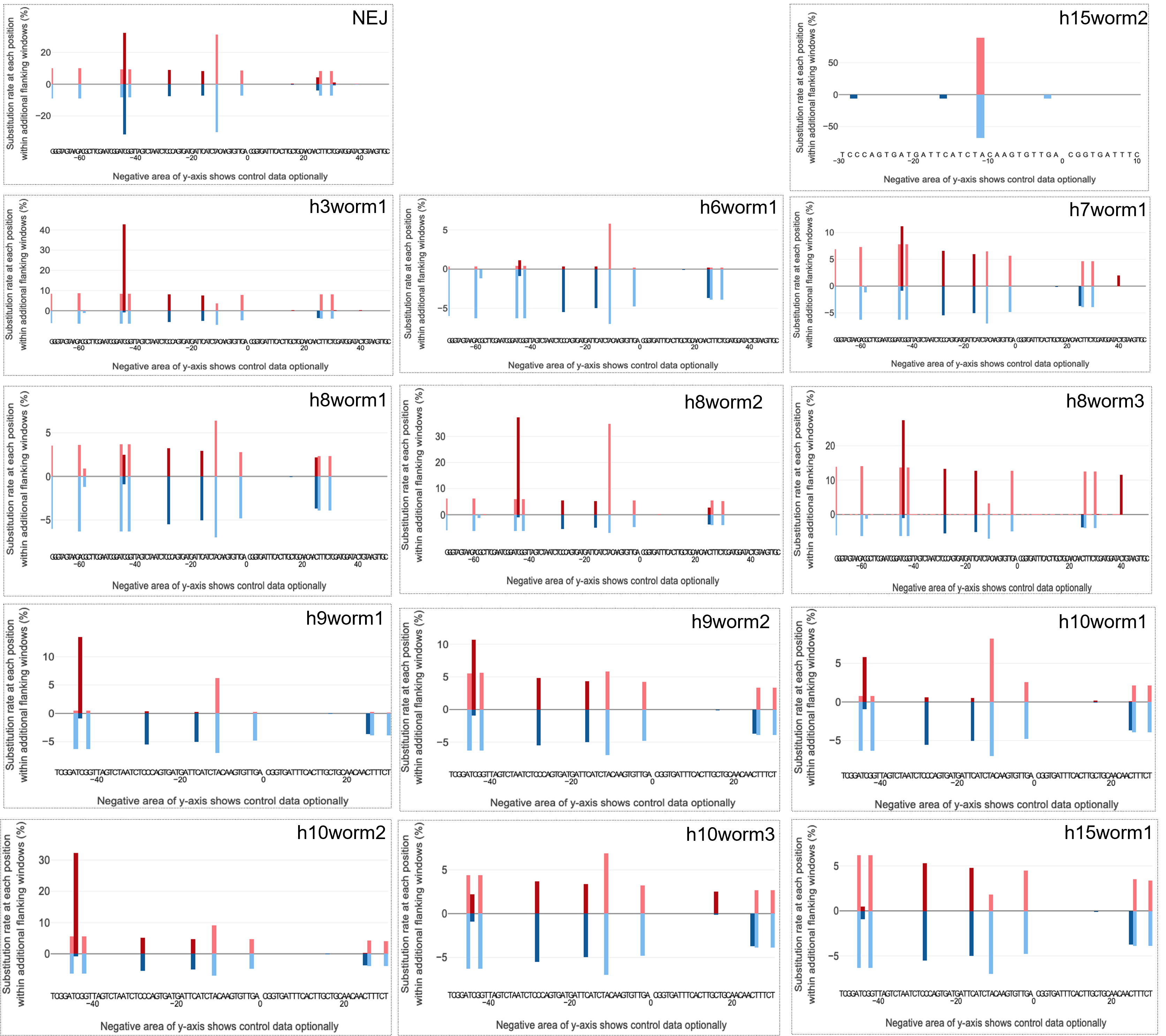
***

**S5 Fig. Profiles of nucleotide substitutions**. Nucleotide substitution profiles detected in the 173 bp amplicon spanning the programmed cleavage site in *Ov-grn-1* from both juvenile (NEJ) and single adult *O. viverrini* flukes of the *ΔOv-grn-1* treatment group compared with the irrelevant guide RNA-treated control group. RGEN’s CRISPR-sub analysis tool, [http://www.rgenome.net/crispr-sub/#](http://www.rgenome.net/crispr-sub/)!, aligns read-pairs to plot the substitution patterns among Illumina sequence reads from amplicon libraries from CRISPR/Cas9 editing-focused datasets. Experimental group (red, upper axis) versus control group (blue, lower axis); the X-axis shows the targeted gene including programmed cleavage site (position 0) between nucleotides 19 and 20 of ORF 1 of *Ov-grn-1*. Juvenile flukes are shown in the top left and each adult fluke is shown separately and designated with the number of its host hamster number (x) 1-15 and worm number (y) 1-3: (h ”x” worm ”y”). Substantial differences were not apparent in patterns of substitutions detected among the experimental and control groups.
