# Supplementary material for: Knockout of liver fluke granulin, Ov-grn-1, impedes malignant transformation during chronic infection with Opisthorchis viverrini
Source: PLoS Pathog. 2022 Sep 22;18(9):e1010839. doi: 10.1371/journal.ppat.1010839 (PMC9531791; doi:10.1371/journal.ppat.1010839)
Supplement: S6 Fig — For Experiment 2, each liver lobe was plotted as eggs per gram against the Ishak fibrosis (data combined from Figs 3A and 6B). Data points have been nudged ± 0.1 on the vertical axis for clarity among overlapping points. The linear regression line for each group is shown; ns, not significant; *, P ≤ 0.05. (DOCX) [file ppat.1010839.s006.docx]

**S6 Fig. Correlation between fecal EPG and fibrosis**. For Experiment 2, each liver lobe was plotted as eggs per gram against the Ishak fibrosis (data combined from Fig 3A and 6B). Data points have been nudged ± 0.1 on the vertical axis for clarity among overlapping points. The linear regression line for each group is shown; ns, not significant; *, *P* ≤ 0.05.
