# Supplementary material for: Knockout of liver fluke granulin, Ov-grn-1, impedes malignant transformation during chronic infection with Opisthorchis viverrini
Source: PLoS Pathog. 2022 Sep 22;18(9):e1010839. doi: 10.1371/journal.ppat.1010839 (PMC9531791; doi:10.1371/journal.ppat.1010839)
Supplement: S1 Table — Frequency of successful CRISPR/Cas9 gene knock out editing as determined by frameshift mutations. The CRISPResso2 analysis used a window size (-3 option) that include the entire 173 bp amplicon, except for 25 bp at each end in order to the exclude the primer binding regions. Combined insertions and deletions are used to generate the % indel, the proportion of read pairs aligned (RPA). To match the color scheme in the figures, the control groups highlighted in blue and low, medium and highly edited adults are highlighted in red, purple and green. Most indels are located in the 5’ UTR and are not frameshift mutations. (DOCX) [file ppat.1010839.s007.docx]

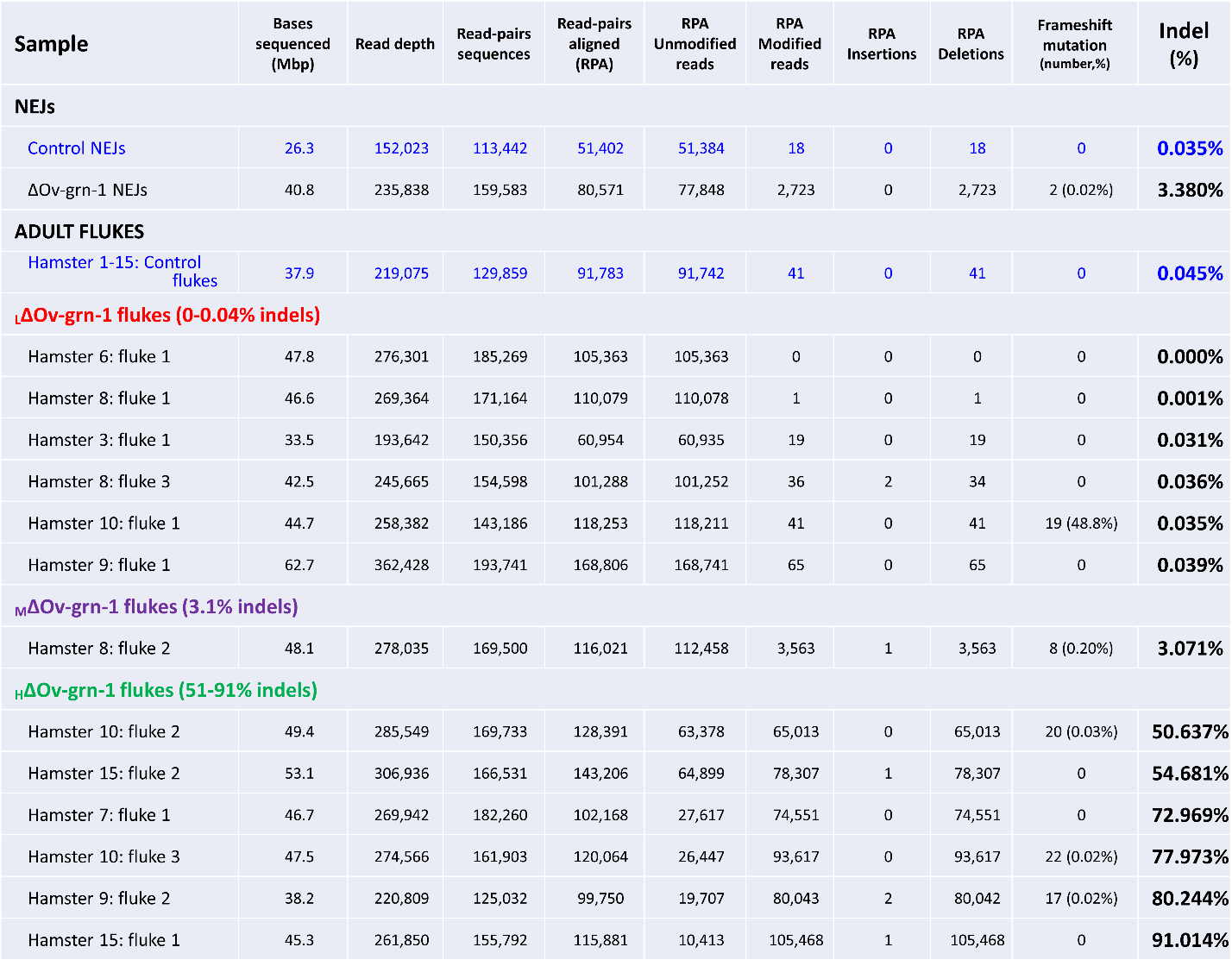


**S1 Table. Summary of NGS data**. Frequency of successful CRISPR/Cas9 gene knock out editing as determined by frameshift mutations. The CRISPResso2 analysis used a window size (-3 option) that include the entire 173 bp amplicon, except for 25 bp at each end in order to the exclude the primer binding regions. Combined insertions and deletions are used to generate the % indel, the proportion of read pairs aligned (RPA). To match the color scheme in the figures, the control groups highlighted in blue and low, medium and highly edited adults are highlighted in red, purple and green. Most indels are located in the 5’ UTR and are not frameshift mutations.
