# Supplementary material for: Knockout of liver fluke granulin, Ov-grn-1, impedes malignant transformation during chronic infection with Opisthorchis viverrini
Source: PLoS Pathog. 2022 Sep 22;18(9):e1010839. doi: 10.1371/journal.ppat.1010839 (PMC9531791; doi:10.1371/journal.ppat.1010839)
Supplement: S2 Table — Disease profiles among the three groups of hamsters and the histopathological diagnosis data was summarized as graphs in Fig 5. Beyond Fig 5, the table outlines the histopathological type of the cholangiocarcinoma (CAA), location, and tumor progression. Representative images of the three major CCA types, A = tubular, B = papillary, and C = mucinous, are included. (DOCX) [file ppat.1010839.s008.docx]

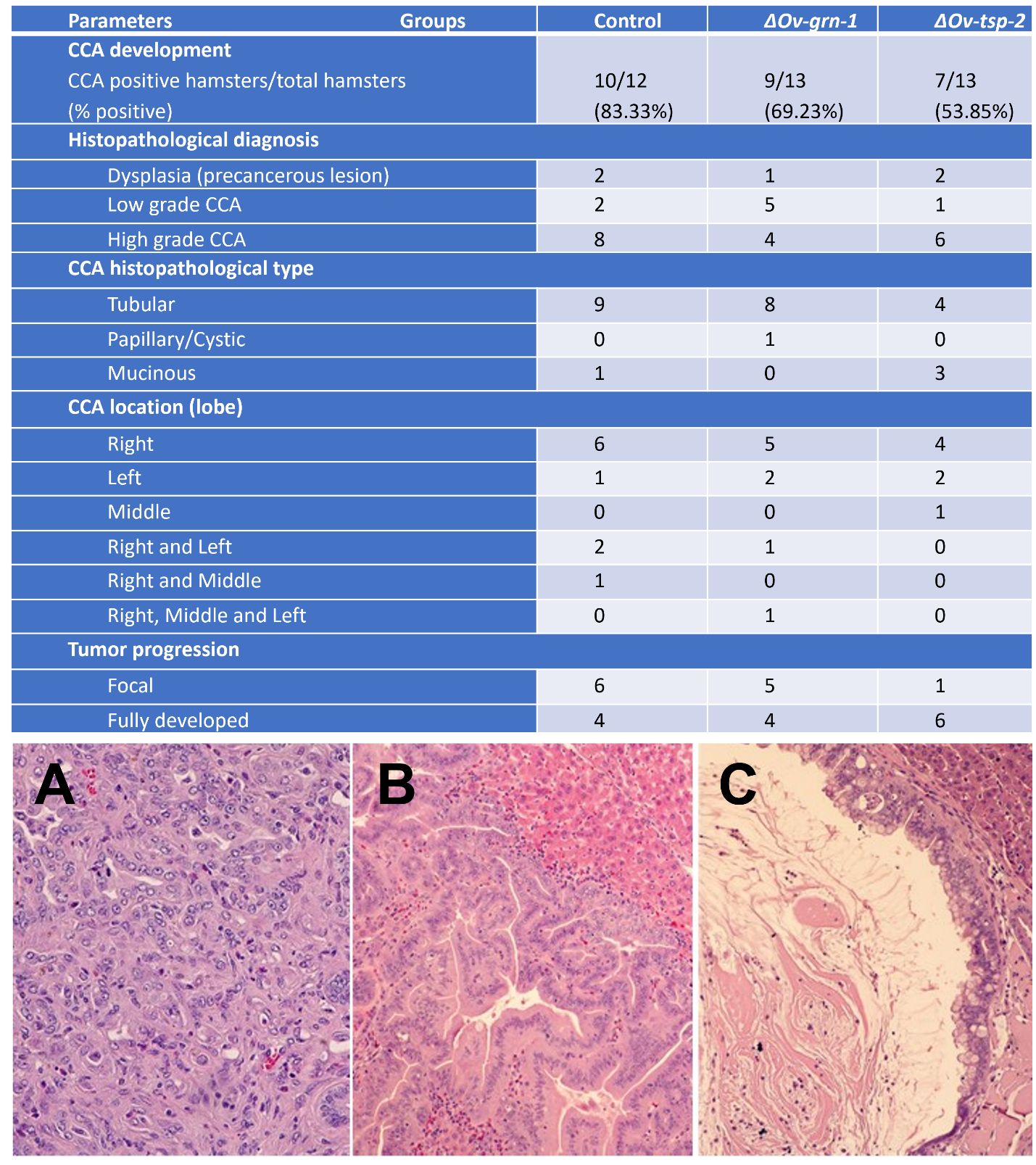


**S2 Table. Assessment of pre-malignant and malignant lesions**. Disease profiles among the three groups of hamsters and the histopathological diagnosis data was summarized as graphs in Fig 5. Beyond Fig 5, the table outlines the histopathological type of the cholangiocarcinoma (CAA), location, and tumor progression. Representative images of the three major CCA types, **A** = tubular, **B** = papillary, and **C** = mucinous, are included.
