# Supplementary material for: Knockout of liver fluke granulin, Ov-grn-1, impedes malignant transformation during chronic infection with Opisthorchis viverrini
Source: PLoS Pathog. 2022 Sep 22;18(9):e1010839. doi: 10.1371/journal.ppat.1010839 (PMC9531791; doi:10.1371/journal.ppat.1010839)
Supplement: Attachment — Submitted filename: PPATH-D-22-0097_Responses 2022-July-27_pjb.pdf [file ppat.1010839.s009.pdf]

27 July 2022

Response to editors and reviewers: Authors' point-by-point responses below in blue.

31 May 2022: "In our opinion, the revision does not require performance of further experiments. Specifically, Reviewer 2 suggested but did not require further experimental work concerning loss of an off-target gene disruption control, but also accepted that the question can be addressed by modification of the text.

Reviewer's Responses to Questions

Part I - Summary
Please use this section to discuss strengths/weaknesses of study, novelty/significance, general execution and scholarship.

Reviewer #1: In this manuscript by Chaiyadet et.al. the investigators used a CRISPR/Cas9 system to knockout a gene in the liver fluke Opisthorchis viverrine and showed that the Ov-GRN-1 gene is important for disease pathogenesis. It should be noted that the ability to genetically manipulate helminth parasites to study pathogenesis has been one of the main limiting factors in the field of helminth biology and hence this represents an important breakthrough for the field, especially for the study of this parasite. While the efficiency of deletion is not ideal, for example in Figure 2 C, there is clearly a bimodal distribution to deletion, with half the flukes having no deletion and the other half having good deletion. Most importantly, the investigators were able to demonstrate that edited parasites had a consequence on fibrosis in an animal model. Mechanistically, this was associated with reduced p53 expression and proliferation of Cholangiocytes, which are the targets for this molecule. This is an important manuscript and a remarkable piece of work by the investigators that is going to have a very high impact on the field of helminth biology.

The authors appreciate and are encouraged by this assessment.

Reviewer #2: Review of MS# PPATHOGENS-D-22-00097 by Chaiyadet, Loukas, Brindley, Laha and others entitled "Programmed knockout mutation of liver fluke granulin, Ov-grn-1, impedes malignant transformation during chronic opisthorchiasis".

This paper is a continuation of ongoing study by the authors of the role of the liver fluke Opisthorchis viverrini in the pathogenesis of human cholangiocarcinoma. Association of O. viverrini infection with this virulent cancer is clear, and preceding studies by this group demonstrated that a secreted tissue repair factor, the granulin encoded in Ov-grn-1, accounts for the majority of the worm's pathogenic effects on the hepatobiliary tract. The most recent findings revealed that disruption of Ov-grn-1 by CRISPR/Cas9, a method developed for parasitic flatworms by the authors, resulted in strong suppression of the gene's transcripts and, concomitantly, of pathologic changes in hepatobiliary tissues surrounding gene-edited worms. This study recapitulates these findings in a study that introduces chronic nitrosamine exposure in the infected hamster model to mimic the contribution of these compounds that would accompany O. viverrini infection by ingestion of preserved fish intermediate hosts. Additionally, the authors

demonstrated that specific indicators of malignant transformation (TP53 mutation and cell proliferation) that are upregulated in infections with wild-type parasites, are suppressed by disruption of Ov-grn-1. These are noteworthy additions to the body of knowledge about O. viverrini infection and cancer.

The authors also appreciate and are encouraged by these comments.

This reviewer notes some substantive shortcomings of the paper, two of which could be dealt with by careful revision of the text. One other, the essential loss of an off-target gene disruption control, may require further experimentation, but at least should be discussed thoroughly by the authors within a revised MS. There are a few minor points of grammar and editing listed as well.

Noted; we have attempted to resolve the shortcomings in the R1 version, as detailed below.

Reviewer #3: This study provides further evidence that the liver fluke protein granulin is involved in the causative pathway to mutagenesis and resulting tumour formation in hamster models. Overall the experiments performed appear to be appropriate and the findings, as claimed, present an advance on current studies. However I raise a number of issues with the presentation of data and the statistical tests performed, which makes it difficult to interpret the study results and assess whether the claimed findings are justifiable.

The authors appreciate and are encouraged by these comments. We have comprehensively and thoroughly addressed the concerns in the R1 version, as detailed below.

Part II – Major Issues: Key Experiments Required for Acceptance
Please use this section to detail the key new experiments or modifications of existing experiments that should be <u>absolutely</u> required to validate study conclusions.

Generally, there should be no more than 3 such required experiments or major modifications for a "Major Revision" recommendation. If more than 3 experiments are necessary to validate the study conclusions, then you are encouraged to recommend "Reject".

Reviewer #1: (No Response)

Reviewer #2: SUBSTANTIVE POINTS

Note: Of these, only point #2 may require additional experimentation, that is assessment of pathogenesis by worms with an off-target gene knockout that allows development of NEJ parasites to the adult stage. Alternatively the authors should thoroughly consider and discuss in the paper the implications of the loss of the Ov-tsp-2 edited controls.

We have addressed the concern using the alternative offered by the reviewer, where in this R1 version we have thoroughly considered and discussed the implications of the loss of the *Ov-tsp-*

2-edited controls, as detailed below.

1. Line 73: readers may be struck by the similarity of this study and its findings to one reported in the 2019 Elife paper (reference 7). It would be helpful at some point, preferably early in the paper, to explicitly state the novel aspects of this study in relation to that previous one. This reviewer assumes the concurrent treatment with nitrosamine is one such additional measure. Is that correct?

Yes, correct, and we have modified the text throughout to differentiate these studies wherever possible.

Also, assessments of cell proliferation and emergence of specific markers of malignant transformation (TP53) appear novel. Also correct?

Yes, correct, and we have modified the text throughout to differentiate these studies wherever possible.

Please elaborate on these and other points to clearly differentiate this paper from the 2019 publication.

We concur that the absence of specific information that differentiates this manuscript from our original paper (Arunsan et al 2019 *eLife*) was a regrettable omission. We now appreciate the opportunity to remedy the problem.

Accordingly, we revised the first paragraph of the **Discussion** to rectify the issue, as follows:

"CCA accounts for ~15% of all primary liver cancers globally and its incidence is increasing (35). Infection with *O. viverrini* is the principal risk factor for CCA in the Lower Mekong River Basin countries including Thailand and Laos PDR, where CCA is the dominant form of liver cancer (1, 22, 36). In an earlier report, we exploited this link to explore the role of *Ov*-GRN-1 secreted by the parasite in tumorigenesis using programmed gene knockout, and reported that the infection was less severe even though gene-edited parasites colonized the biliary tract of hamsters and developed into adult flukes (18). In this follow-on investigation, we report findings during concurrent exposure to dietary nitrosamine and infection with the gene knockout parasites, and that KO of the granulin gene retards malignant transformation to CCA, including the emergence of mutant p53, in a rodent model of human opisthorchiasis-associated CCA. These novel results build upon and advance the findings from our original report and, centrally, confirmed the role of liver fluke granulin in malignant transformation during chronic opisthorchiasis".

The notable difference between the earlier paper and this manuscript, and the novel and noteworthy advance that we report here, is the assessment of malignant transformation and development of cholangiocarcinoma. To enable this advance, here (unlike the 2019 report) we utilized a hamster model of infection-induced malignancy rather than simply infection associated biliary hyperplasia and hepatic fibrosis. In addition, as pointed out by the reviewers, we observed

and now report the remarkable reduction in cancer development in the *Ov-grn-1* KO flukes compared to *Ov-tsp-2* KO flukes or control flukes.

2. Line 137 and in general: it's not clear to this reviewer why Ov-tsp-2 was selected as a control gene for knockout. Was it simply a non-target gene against which to compare phenotypes resulting from OV-tsp-2 knockout? If so, it's unclear why a gene that's essential for normal development in other parasitic flatworms, such as schstosomes, would be selected. Ultimate lethality of Ov-tsp-2 knockout was borne out by the data indicating that no or few Ov-tsp-2 knockout worms survived to the adult stage in the infected hamsters. That would seem to diminish or eliminate the value of these Ov-tsp-2 knockouts as controls. The authors should discuss this point, evaluating the impact of losing this control on the integrity of the study.

We have addressed this issue in each of the Introduction, the Discussion, and the Materials & Methods sections, as follows:

Introduction

"…For this investigation, hamsters were infected with juvenile flukes that had been genetically modified using CRISPR before infection. Specifically, following up on our earlier study (18) which focused on knockout of the *Ov-grn-1* gene, here we included as comparators juvenile flukes gene-edited for a second virulence factor, tetraspanin (*Ov-tsp-2*) (23), which also networks at the host-parasite interface (24, 25) and, as controls, flukes subjected to CRISPR transfection by an irrelevant (non-targeting) guide RNA (26)".

Discussion (paragraphs 3 and 4):

"Fitness cost of gene knockout can be assessed from programmed gene editing, an approach that is employed for the unbiased identification of essential genes in other organisms and disease settings (40, 41). The present findings confirmed the power of RNA-guided targeted mutation to define essentiality and relevance of two parasite proteins in infection-associated morbidity and malignancy. The *Ov-grn-1* gene does not appear to be essential for *in vivo* development and survival, which has enabled investigation here on the role of this protein in driving cell proliferation, pathology and ultimately contributing to CCA. Nonetheless, the reduced fecundity of *ΔOv-grn-1* liver flukes likely reflected a fitness deficit as the result of the targeted KO. By contrast, *Ov-tsp-2* appears to be essential to parasitism. The *ΔOv-tsp-2* genotype did not survive *in vivo*, and sequencing of the indels across the relevant region of the genome confirmed that most of the surviving flukes from hamsters had not undergone editing at the *Ov-tsp-2* gene locus. These findings build upon earlier RNA interference-mediated silencing of *Ov-tsp-2* gene expression and the resultant malformation of the tegument observed *in vitro* (42). Although infection of hamsters with *Ov-tsp-2* dsRNA-treated parasites was not investigated in this earlier report, the damage to the tegument following exposure to *Ov-tsp-2* dsRNA in worms cultured for several days appeared to be so extensive and debilitating that worms damaged to that extent by either CRISPR-based genome knockout or dsRNA likely did not establish or survive for long periods *in vivo*.

In *O. viverrini*, *Ov*-GRN-1 and *Ov*-TSP-2 share key, though dissimilar functions at the parasite interface with the mammalian host. *Ov*-GRN-1 induces proliferation of cholangiocytes whereas *Ov*-TSP-2 is a key structural protein of the tegument of liver flukes (and indeed in schistosomes (43, 44)) and of extracellular vesicles that are taken up by host cholangiocytes, among other roles (15, 16, 24). Accordingly, *Ov-tsp-2* was included here as a comparator gene for *Ov-grn-1* KO. A non-targeting guide RNA encoded here by pCas-*Ov*-scramble, also was included, to provide a negative control for off-targeting by the Cas9 nuclease (26). Ultimate lethality of *Ov-tsp-2* KO was borne out in our observation that *ΔOv-tsp-2* genotype flukes failed to survive to the adult stage in the hamsters. Whereas this diminished the value of *ΔOv-tsp-2* worms as controls, the findings highlighted the apparent essentiality of this tetraspanin to the intra-mammalian stages of the liver fluke. This essentiality of tetraspanin contrasted with *Ov*-GRN-1, the absence of which was not lethal to the parasite but which, fortunately, enabled inferences on its contribution to malignant transformation and which reinforced earlier hypotheses on its role as a carcinogen (15). These approaches and findings are novel in the field of functional genomics for helminth parasites and hence, even if targeting *Ov-tsp-2*, in hindsight, was a misstep, the outcome provided a programmed mutation-based demonstration of the essentiality and lethality of mutations of these liver fluke genes, which represents vanguard progress in forward genetics for helminth parasites. Indeed, for context concerning the pioneering significance of this advance, establishing the essentiality of human genes is an active and fertile field in functional genomics and gene therapy (45, 46)".

Materials & Methods

"The CRISPR plasmid encoding a guide RNA (gRNA) complimentary to *Ov-grn-1* exon 1 termed pCas-*Ov-grn-1* was constructed using the GeneArt CRISPR Nuclease Vector kit (Thermo Fisher Scientific), as described . The programmed cleavage site at nucleotide position 1589–1608, 5'-GATTCATCTACAAGTGTTGA with a CGG proto-spacer adjacent motif (PAM) which determined the cleavage site located at three nucleotides upstream on *Ov-grn-1* was designed using the online tools, http://crispr.mit.edu/ (68) and CHOPCHOP, http://chopchop.cbu.uib.no/ (69, 70) using the *Ov-grn-1* gene (6,287 bp, GenBank FJ436341.1) as the reference. A second plasmid, termed pCas-*Ov-tsp-2* was constructed using the same approach; pCas-*Ov-tsp-2* encodes a gRNA targeting exon 5 of the *Ov-tsp-2* gene (10,424 bp, GenBank JQ678707.1) (71, 72). The guide RNAs encoded by *Ov-grn-1* and *Ov-tsp-2* exhibited theoretically high, on-target efficiency and little or no off-target matches to the *O. viverrini* genome. A third construct, termed pCas-*Ov*-scramble was also prepared and included as a control to normalize analysis of gene expression and programmed gene knockout. The pCas-*Ov*-scramble construct included as the gRNA, a transcript of 20 nt, 5'-GCACTACCAGAGCTAACTCA which exhibits only minimal identity to the *O. viverrini* genome and which lacks a PAM (73). A non-targeting guide RNA (26), encoded here by pCas-*Ov*-scramble, provided a negative control for off-targeting by the Cas9 nuclease".

3. Line 536-543: Given this observation, readers may question the advantages of targeted gene mutation via CRISPR/Cas9 over previous RNAi based approaches to functional genomics in parasitic flatworms, which also resulted in significant, but not complete ablation of target gene

transcripts and, in studies of putative virulence factors, incomplete resolution of lesions. Can the authors comment on this in light of previously published work?

RNAi has been widely used to knock down various flatworm life cycle stages *in vitro*, including by us with both *Ov-grn-1* and *Ov-tsp-2*. Our concern over using RNAi was the lengthy *in vivo* period (24 weeks) required for this experiment during a phase of rapid growth from the NEJ to the adult. This was demonstrated in a recent report dealing with *Fasciola hepatica* in which the RNAi effectiveness was dramatically reduced over a single week, and hence deployed, retreatment of RNAi oligos every 3-4 days (McCusker *et al* 2020 PMID: 32866764). Moreover, retroviral systems can be used to counter RNAi dilution from cell division in schistosomes (e.g., Hagen et al 2014 PMID: 25400038) nonetheless, however, we concluded based on our 2019 *eLIfe* report that altering the genome of the parasite would be a appropriate and permanent approach to ablate gene expression in our investigation and facilitate clinical observations *in vivo* over many months to facilitate malignant transformation and manifestation of cholangiocarcinoma.

Reviewer #3: None

Part III – Minor Issues: Editorial and Data Presentation Modifications
Please use this section for editorial suggestions as well as relatively minor modifications of existing data that would enhance clarity.

Reviewer #1: (No Response)

Reviewer #2: MINOR POINTS OF EDITING AND GRAMMAR

4. Line 40: suggest inserting "(CCA)" after "cholangiocarcinoma". Done.

5. Lines 70-71: consider "diseases of the liver and bile ducts" rather than "hepatobiliary diseases" for lay readers. Revised as suggested.

6. Line 91: this sentence might read more smoothly if you made it "….CCA are…" rather than "…CCA is..". Revised as suggested.

7. Line 105: consider "promotes" rather than "can promote". Revised as recommended.

8. Line 109-110: suggest deleting ", shown" after "is feasible and". Revised as suggested.

9. Line 113: perhaps "the pathogenesis of CCA", rather than "induction of CCA"? Revised as recommended.

10. Line 119-123: this is a wordy and cumbersome sentence as written. Consider revising for conciseness and clarity.

Revised for clarity and length, as follows:

"In the hamster, chronic opisthorchiasis provokes periductal fibrosis, which, coincident with exposure to the nitric oxide carcinogen facilitates cholangiocyte proliferation, epithelial hyperplasia, DNA damage, and allied biliary tract lesions (21), which can culminate in CCA (22)".

11. Line 128: consider "highlighted" rather than "highlight". Revised to "highlighted".

12. Line 187: substitute "did not show" for "not showing. Substituted "did not show" for "not showing".

13. Line 188: likewise, substitute "showed" for "showing". Likewise done.

14. Line 223: make it "fecal egg counts". Revised to "fecal egg counts".

4. Line 286: delete "a" after "showed". Deleted.

5. Line 388: need a comma after "infection". Comma inserted.

6. Line 413: make it "different from the control group". Revised to "different from the control group".

7. Line 426: suggest deleting "as a consequence". Deleted the phrase.

8. Line 621: The designation Syrian golden hamsters, Mesocricetus auratus should be moved to the first reference to hamsters in Materials and Methods.

Designation has been revised as recommended such that the fist mention is in the Materials and Methods, sub-section "**Infection of hamsters with gene edited *O. viverrini* juveniles"**

Figure 1 provides a timeline of the CCA model, employed in Experiments 1 and 2. In a first experiment, nine male hamsters (Syrian golden hamster, *Mesocricetus auratus*) aged between 6-8 weeks were randomly divided into three experimental groups (Figure 1)."

9. Line 663: Suggest "Feces from each hamster were…". Revised as suggested.

10. Line 793: Shouldn't it read "P<0.05"? Corrected as "$P \leq 0.05$".

11. Line 833 and following: see to italicizing genus/species names in the bibliography.

Will italicize genus/species names in bibliography and interact with the PLOS publishing team at the point of type setting to conform with the journal's style.

Reviewer #3: These comments are given in the order at which they appear in the text, rather than in order of importance:

12. The article title should be revised, "programmed knockout mutation" seems redundant and is identical to the title of a related article by the same authors; "Programmed knockout mutation of liver fluke granulin attenuates virulence of infection-induced hepatobiliary morbidity" (Arunsan 2019 Elife). Consider instead "Knockout of liver fluke granulin, Ov- grn-1 , impedes malignant transformation during chronic infection with Opisthorchis viverrini"

Title revised as recommended to "Knockout of liver fluke granulin, *Ov- grn-1* impedes malignant transformation during chronic infection with *Opisthorchis viverrini*"

And the Short Title revised to "Knockout of liver fluke growth factor impedes bile duct cancer".

13. Abstract lines 46-49 "Whereas Ov-grn-1 gene-edited parasites colonized the biliary tract and developed into adult flukes, less hepatobiliary tract disease manifested during chronic infection with ΔOv-grn-1 worms in comparison to hamsters infected with control parasites". This doesn't make sense to me. Do the authors mean instead "Gene-edited parasites colonized the biliary tract and developed into adult flukes, however less hepatobiliary tract disease manifested during chronic infection with ΔOv-grn-1 worms in comparison to hamsters infected with control parasites" ?

Revised to the text recommend by the reviewer. With thanks.

14. Introduction lines 91-94: the impact of nitrosamines in fermented foods on carcinogenesis needs a citation.

Two citations added in support of the statement:

Srivatanakul P, Ohshima H, Khlat M, Parkin M, Sukaryodhin S, Brouet I, et al. *Opisthorchis viverrini* infestation and endogenous nitrosamines as risk factors for cholangiocarcinoma in Thailand. Int J Cancer. 1991;48(6):821-5.

Sriraj P, Boonmars T, Aukkanimart R, Songsri J, Sripan P, Ratanasuwan P, et al. A combination of liver fluke infection and traditional northeastern Thai foods associated with cholangiocarcinoma development. Parasitol Res. 2016;115(10):3843-52.

15. Introduction lines 91-94: It is unhelpful and somewhat lazy to repeat the mantra that "The mechanism(s) by which opisthorchiasis leads to CCA is likely multi-factorial..." after the amount of evidence accumulated in this area. Some factors are invariably more important than others and their impact can be quantified. In your previous study (Arunsan 2019 Elife) what proportion of the variance in mutagenesis was explained by knockdown of Ov-grn-1? How much of the

variance can be explained by differential worm burdens? The authors should be attempting to quantify the relative importance of these different exposures in their experimental system.

We revised the offending text and now include comment in Introduction on the role of some of the factors, second paragraph of the Introduction. (This issue is also addressed in the Discussion).

"Which features or consequences of parasitism by the liver fluke definitely initiate malignant transformation to CCA have yet to be ascertained notwithstanding that opisthorchiasis is the principal risk factor for CCA in regions where this neglected tropical disease remains endemic (1, 4-6). Some factors can be expected to more important than others and the impact of these factors should be quantifiable. Different worm burdens play a role, based on rodent models of liver fluke infection associated CCA (7), as does concurrent exposure to nitrosamines in fermented foods (8, 9) that are culturally important dietary staples in countries of the Lower Mekong River basin (5). Moreover, dose-dependent, synergistic effects of the liver fluke and nitroso-compounds have been documented (7, 10). To survive within the host, parasitic helminths actively release excretory/secretory (ES) proteins and other mediators with diverse effects and roles at the host-parasite interface (11, 12). This interaction is considered to manipulate host cellular homeostasis and, moreover, to underpin malignant transformation during chronic opisthorchiasis, but the molecular mechanisms by which these processes remain inadequately understood (13)".

16. Introduction lines 106-8: "Also, we recently confirmed the role of Ov-GRN-1 in driving proliferation of bile duct epithelial cells (cholangiocytes) by genetic manipulation of its expression in the liver fluke both by RNAi and by CRISPR/Cas9 gene editing (7)." The cited study (Arunsan 2019 Elife) appears similar in content to this manuscript. There needs to be additional explanation for the limitations in previous work which are overcome by the present study and what these experiments add to current understanding.

The Discussion has been revised to address this recommendation, as follows:
Discussion

""CCA accounts for ~15% of all primary liver cancers globally and its incidence is increasing (35). Infection with *O. viverrini* is the principal risk factor for CCA in the Lower Mekong River Basin countries including Thailand and Laos PDR, where CCA is the dominant form of liver cancer (1, 22, 36). In an earlier report, we exploited this link to explore the role of *Ov*-GRN-1 secreted by the parasite in tumorigenesis using programmed gene knockout, and reported that the infection was less severe even though gene-edited parasites colonized the biliary tract of hamsters and developed into adult flukes (18). In this follow-up investigation, we report findings during concurrent exposure to dietary nitrosamine and infection with the gene edited parasites, and that KO of the granulin gene retards malignant transformation to CCA, including the emergence of mutant p53, in a rodent model of human opisthorchiasis-associated CCA. These novel results build upon and advance the findings from our original report (18) and, notably, confirmed the role of liver fluke granulin in malignant transformation during chronic opisthorchiasis (15). We utilized an established model of opisthorchiasis-associated CCA in hamsters that were infected with the parasite during concurrent exposure to exogenous

nitrosamine. CCA manifests under these conditions, and this rodent model reflects the human situation where chronic opisthorchiasis in the context of a diet that is rich in fermented fish (in turn, rich in nitrosamines) culminates in a high incidence of CCA (20, 22, 37, 38). In hamsters, opisthorchiasis leads to periductal fibrosis. Chronic periductal fibrosis combined with a nitric oxide carcinogen, such as DMN, results in epithelial cholangiocyte proliferation, hyperplasia, dysplasia, and DNA damage, eventually and reliably manifesting as malignant neoplasia of the biliary tract (20, 21, 39). By contrast, conspicuously less proliferation of the biliary epithelium, reduced mutant p53 expression by cholangiocytes, and less periductal fibrosis accompanied infection here with *ΔOv-grn-1* genotype worms compared to controls. As noted, our approaches and findings represent a functional genomics (forward genetics)-focused extension of the model pioneered by Thai investigators more than 30 years ago (7).
".

17. Results line 135: The abbreviation of "newly excysted juveniles (NEJs)" adds to a number of acronyms in the paper and is unlikely to be familiar to many readers. Consider replacing this acronym with "juveniles" throughout.

We have revised the Materials and Methods in response to and to address this point, as follows:

"Newly excysted juveniles (NEJ) of *O. viverrini* were induced to escape from the metacercarial cyst by incubation in 0.25% trypsin in PBS supplemented with 2× 200 U/ml penicillin, 200 µg/ml streptomycin (2× Pen/Strep) for 5 min at 37°C in 5% CO_2 in air. The juvenile flukes were isolated free of discarded cyst walls by mechanical passage through a 22 G needle (18). We also use the term NEJ for the juvenile flukes because NEJ is also widely used for juveniles of related liver flukes (66, 67)".

The term "NEJ" has frequently been used by other authors in their papers in *PLoS Pathogens*, including in Ref. 67, above. Nonetheless, to address the reviewer's concern, we have also generally replaced "NEJ" with for "juvenile flukes" throughout most of the R1 version.

18. Results line 137: This is the first mention of Ov-tsp-2. What is the function of this gene and why is it being knockout out in addition to Ov-grn-1?

Please see response to Reviewer no. 2, item 2.

19. Results line 137: The use of "control" throughout, rather than "SCR" (scramble) would improve readability of the manuscript

We revised the manuscript, entirely replacing "SCR" (scramble) with "control" with the exception of the name of the plasmid encoding the irrelevant guide RNA.

20A. Figure 1A: This figure could certainly be improved for clarity. Consider revising the tubes and lighting bolts on the left hand side. There are no images of O. viverrini worms, which makes it seem like the hamsters are being gene edited rather than the parasites.

Figure 1 has been revised for clarity, including with the addition of the lightning bolts on the left to illustrate that flukes (not hamsters) were the focus of the programmed gene knockout.

20B. The rationale for the two experiments are also unclear at this stage.

Experiment 1 assessed worm burdens. Experiment 2 assessed pathology and, accordingly, to avoid damaging the issues at necropsy by recovering the liver flukes from the bile ducts (a destructive procedure), precise assessment of the numbers of worms parasitizing each hamster was not feasible. The Results section has been revised to explain the rationale and the clarify the procedures. This is the rewritten, opening paragraph of the Results section of the R1 manuscript, wherein we clearly explained the rationale for and differentials the two experiments:

"**Hamster model of malignant transformation during infection with gene-edited *Opisthorchis viverrini***

To investigate the effect of programmed gene knockout (KO) in *O. viverrini*, two experiments were undertaken in which hamsters were infected with newly excysted juveniles (NEJs) of *O. viverrini* that had been subjected to programmed KO . The CRISPR/Cas systems were delivered by electroporation of plasmids encoding guide RNAs specific for *Ov-grn-1*, *Ov-tsp-2* and an irrelevant (control) guide RNA. All groups received plasmids encoding the Cas9 nuclease from *Streptococcus pyogenes*. Figure 1A illustrates the experimental approach and timelines, the findings from which we present below. Initially, juvenile flukes were subjected to transfection after which reduction in transcription of the targeted genes, *Ov-grn-1* and *Ov-tsp-2*, was verified. Subsequently, following successful KO of transcription *in vitro*, additional juvenile *O. viverrini* were transfected before infection of hamsters. The goal of Experiment 1 was to assess impact of KO on the worm burden. At necropsy14 weeks after infection, the entire liver was examined, the worms were recovered and counted, and transcriptional changes were investigated in the worms. With Experiment 2, the primary goal was establishment and assessment of disease burden including malignant transformation during infection. Given that livers from the hamsters in Experiment 2 were fixed at necropsy, worm burdens could not be established directly because recovery of the flukes is a process that damaged the liver and biliary tract and, accordingly, was incompatible with histological examination of infection-associated disease. Consequently, only a small number flukes were available from Experiment 2 and these that were available were collected incidentally during preparation of the liver lobes for fixation. Nonetheless, this sample of the flukes from each of the three groups was sufficient to assess the performance and level of programmed KO although total worm burden was unavailable. Nonetheless, the findings from Experiment 2 provided the first description of gene-edited flukes in chronically-infected hamsters, i.e., with duration of infection beyond eight weeks and, additionally, the first time programmed mutation of the liver fluke genome has been combined with exposure to dietary nitrosamine in the hamster-liver fluke model of human CCA".

21. Figures 1B and C: There are a number of issues with both this figure and the analysis of these data. Presented as a "percentage of control abundance" is confusing as the SCR samples themselves vary. Presumably "100% of control" is the mean abundance in the SCR group? The following recommendations are: A) present the data on the original scale (number of transcripts), B) show each data point individually, rather than as a bar chart, C) remove the references to

significance testing (stars and NS), D) replace the label "SCR" with "control", E) remove "NEJ" text on y axis.

We have endeavored to incorporate the reviewer's points but we consider that presenting the data on a percent scale provides the clearest communication of the transcript reduction to a wide variety of readers.

A) As these were data from qPCRs, the number of transcripts was not available. However, we now provide a supporting figure with the ddCt scores with bootstrapped averages and 95% confidence intervals (Figure S1).
B) Each point was now shown individually with the average as a colored line.
C) We removed significance testing of the qPCR data
D) As suggested, control has now been used instead of SCR throughout the report
E) NEJ has been removed from the axis

22. Relating to this, the statistical tests performed to demonstrate gene knockout are unclear and likely inappropriate. Was the "one-way ANOVA with Holm-Sidak multiple comparison" performed on the original transcript abundance data, or after transformation into a relative proportion? I would strongly recommend against transforming the data prior to analysis. The ANOVA results (effect size) are also not shown. Given the small number of samples it would be better to use a statistical procedure other than ANOVA. Please calculate 95% confidence intervals within each experimental group by bootstrapping the transcript abundance data and check for overlap. The 95% confidence intervals can then be overlaid over the data points in Figures 1B and C, as recommended above.

In like fashion to our response to Q21, we removed statistics on qPCR data and provided a supporting figure with the ddCT transcript data with bootstrapped averages and 95% confidence intervals. As noted, we consider that presenting the data on a percent scale provides the clearest communication of the proportions of transcript reduction and is a widely used format in other peer-reviewed reports.

23A. Figures 2A and 2B, show all data points on the plots and remove text relating to significance testing (stars and NS).

For Figure 2A, B, dealing with EPG and worm burden data of Experiment 1, all the data points were shown. We consider that the statistical analysis is an important aspect of this analysis. It was important to emphasize that EPG did not change significantly or substantially although the worm burden was significantly reduced in both gene knockout worm groups compared to the controls. We appreciate the reviewer's concerns regarding a singular focus on statistical relevance; however, in this instance, indicating the level of significance is informative.

23B: How are the values in Figures 2C and D different to Figures 1B and C? According to the text, these are all results from "experiment 1". Please show the raw data points rather than violin plots. Again I ask to authors to calculate 95% confidence intervals by bootstrapping values of untransformed transcript abundance.

Figures 1B, C provide transcript levels in juveniles (NEJs) before hamster infection, while Figures 2C, D provide transcript levels in adult flukes at 14 weeks. Additional details have been included to improve clarity, notably in Figure 1 and expanding details of the rationale for the work in the opening paragraph of the Results section. Please also refer to our response to Q20b, above. As requested, all qPCR data has been updated with individual transcript ddCT values plotted as supporting figures with 95% bootstrapped values of confidence intervals.

24. Results lines 183-4: "183 Transcript levels of both genes expressed by the control SCR parasites were generally clustered 184 around 100% (Figure 2C, D)." Again, what is 100%? Is this the mean transcript abundance in the SCR group? In which case it's unsurprising that the relative values in this control group would be close to the mean. Instead provide the variance in transcript abundance on an untransformed scale.

We apologise for this omission and confusion. The “100%” on the axis of Figure 2C, D was the transcript level of wild-type flukes and provided the rationale for describing the control fluke expression levels clustering around 100%. The figure axis and Results text have been revised to specifically mention “wild-type” to clarify the 100% value. Additionally, a supporting figure has been included with the ddCT transcript levels and 95% confidence intervals generated from bootstrap analysis (see Q21).

25. Results line 187-9: "The outcome, where most flukes either not showing a change (~100%) or, by contrast, showing a near absence of transcription (~0%) was not normally distributed, which required a non-parametric statistical approach". This does not make sense. The parametric distribution for an overdispersed proportion is the beta-binomial; there is no need to use non-parametric methods. As mentioned before, the counts of transcript abundance should not be transformed into relative proportions prior to analysis. Until this analysis is re-performed, the changes in transcript abundance given in the text are difficult to interpret.

As noted above, statistics have been removed from qPCR data and replaced with 95% CI bootstrapped values, and the raw ddCT data provided in supporting figures.

26. Results line 196-7: Is it a "lethal phenotype" if 1/3 of knockout worms survive?

Given that a multicellular, macroscopic worm was the target of the programmed gene knockout, mosaicism was the not unexpected outcome of the somatic gene editing. Indeed, there were worms that expressed *Ov-tsp-2*. We now posit that that efficient KO of *Ov-tsp-2* and consequently ablation of *Ov-tsp-2* expression was lethal, and accordingly worms that did not express *Ov-tsp-2* were not seen because they failed to survive in vivo. The majority of flukes recovered at 24 weeks showed minimal to no gene KO and transcript levels similar to wild-type flukes (Figures 2D: 82.5% and 3D: 98.5% bootstrap means). The lowest level of *Ov-tsp-2* transcription was 36.6%. This contrasted with *Ov-grn-1* where a broad range in expression levels was seen, and where many worms do not express the transcript (Figs 2C, 3C; 21 worms - < 20% and 10 worms < 2%, with bootstrap mean values of 11.8% and 10.6%). Accordingly, surviving *Ov-tsp-2* edited worms had undergone minimal to absence of gene editing.

27A. Results line 223-22: "In lieu of counting the number of worms parasitizing each hamster, fecal egg count (as eggs per gram of feces, EPG) were determined at the time of necropsy. Numbers of eggs of O. viverrini positively correlate with number of worms within the hepatobiliary tract (12, 13)". The citations given here are both inappropriate as they relate to human studies (and one is in Clonorchis), rather than an experimental hamster system.

We have extensively edited this section and no longer emphasize data in terms of EPG. The revised Results are provided in lines 209 to 300 of the R1 version and include Figure 2.

27B. The authors have the data to show the relationship between egg counts and worm burden from experiment 1 (from Figure 1: EPG values 1 and 2, and worm counts at necropsy). Please plot these data (egg counts vs worm burden) and calculate the relationship between them - note that the relationship may be non-linear due to density dependent fecundity (eggs per worm decrease at high worm burdens) and so the analysis may require a non-linear function (e.g. power law).

No substantial relationship between EPG and worm burden at either timepoint was detected; see additional supporting information, Figure S2. Linear regression of each timepoint was not different from the zero slope (flat line) at either 10 ($p=0.30$) or 12 weeks ($p=0.76$). No Other relationships were not apparent.

28. Results lines 232-238: "Hence, to mitigate the impact of variations in numbers of liver flukes on the analysis and interpretation of the role of gene knockout on hepatobiliary disease and malignancy, the histopathological assessments focused on the livers of hamsters with "moderate" EPG values, i.e. ΔOv-grn-1 EPG ranging from 1,000 to 20,000 (Figure 3A, B, dotted lines). This cutoff window excluded three SCR hamsters with values of 26,000 to 44,000 EPG and four ΔOv-tsp-2-infected hamsters with ≤238 1,000 EPG". This is completely inappropriate. The statistical analysis should correct for the number of parasites (either EPG or inferred worm burden given the relationship estimated above) in a multiple regression model. This would have the advantage of disentangling the effect of pathology due to worm burden versus knockdown of granulin. Excluding animals with arbitrary cutoffs of EPG reduces the power of the analysis, is unethical given the hamsters have been euthanised, and has no basis in experimental design.

We concur. We have revised the manuscript extensively to remove our original focus only on the "moderate EPG" phenotype. All hamsters are included in the revised analyses.

29A. Results 279-281: "Substitution patterns as determined by the CRISPR-sub tool (15) in reads in the knockout groups were not significantly different from the cognate alleles in the control SCR group worms (Figure S1)." This supplementary figure is confusing and does not substantiate the claim that read depth is the same across study groups. Please clarify the y-axis as a value cannot be both a rate and a percentage.

The legend for the figure, which is the R1 version now is Figure S5, has been revised for clarity, and to correct the rate/percentage error.

29B: The x-axis is very unclear - which region of the gene / genome is this?

The region analysed was an amplicon of 173 bp in length spanning the programmed double stranded break at nucleotides 19/20 of ORF 1 of *Ov-grn-1*. This point has been clarified in the legend text.

29C: The authors should instead show read depth plots from a genome browser with variants marked, and report the number of mapped reads in the region of interest across samples.

Regarding read depth for both substitutions and indels, read depth has minimal impact on the analysis as each sample was sequenced deeply with >50,000 aligned read-pairs. Confusion may have arisen because of the difference of whole genome sequencing analysis where read depth is critical in contrast to the current study where a short amplicon (rather than the whole genome) was sequenced and analysed to enable complete aligned read pairs. The CRISPResso and BE analyser tools accept only "read pairs aligned" (and do not accept out partial reads) to estimate indel and substitution rates. However, for clarity, additional columns for both "bases sequenced" and "read depth" have been included in Table S1. Also, the text was revised to replace "reads" with "aligned read-pairs" when discussing mutation rates.

The revised legend (Figure S1 of the original is now Figure S5 in the R1 version) reads:

"Figure S5. Profiles of nucleotide substitutions. Nucleotide substitution profiles detected in the 173 bp amplicon spanning the programmed cleavage site of *Ov-grn-1* from both juvenile (NEJ) and single adult *O. viverrini* flukes of the *ΔOv-grn-1* treatment group compared with the irrelevant guide RNA treated control group. RGEN's CRISPR-sub analysis tool, http://www.rgenome.net/crispr-sub/#!, aligns read-pairs to plot the substitution patterns among Illumina sequence reads from amplicon libraries derived from CRISPR/Cas9 editing-focused datasets. Experimental group (red, upper axis) versus control group (blue, lower axis); the X-axis shows the targeted gene including programmed cleavage site (position 0) between nucleotides 19 and 20 of ORF 1 of *Ov-grn-1*. Juvenile flukes are shown in the top left and each adult fluke is shown separately and designated with the number of its host hamster number (x) 1-15 and worm number (y) 1-3: (h "x"worm"y"). Substantially differences in patterns of substitutions detected between the experimental and control groups were not apparent."

30. Results 284-285: "The ΔOv-grn-1 pooled NEJs showed 3.26% indel levels (2,723 of 80,571 reads), significantly 285 more than the 0.035% level in the SCR NEJ group (18 of 51,402 reads) ($P \leq 0.05$)." This does not make sense. Indels are reported as a function of read depth per sample. Please demonstrate that indels have been called correctly and give the proportion of knockdown samples with indels in the gene regions of interest, rather than the proportion of reads.

As with query 29, this misunderstanding may have arisen from our use of the term "reads" when referring to "aligned read-pairs". The analysis used the complete 173 bp amplicons (aligned read

pairs) for the mutation analysis. The R1 version has been revised to use the term "aligned read-pairs". In addition, for completeness, "read depth" has been added to Table S1.

We used the control group NGS of the amplicon of interest for comparisons with the treatment groups. Only minor differences were observed in the control group from the expected wild-type sequence (NEJs 0.035% indels and adults 0.045% indels). Accordingly, we consider that if bases were miscalled, this occurred infrequently.

31. Results line 298: 3.1% of what?

3.1% indels were detected in the aligned read pairs of the sequence reads. The revised text now states, "These ranged from an apparent absence of programmed mutation (no indels) to near complete KO (91% indels), with a median of 3.1% indels ($_{M}\Delta$*Ov-grn-1*), which was significantly higher than in the control group flukes ($P \leq 0.01$)".

32: How are indels in knockout worms assessed if the adult flukes cannot be recovered from euthanised hamsters?

Some KO flukes (a small number of the fluke population) were recovered: fortuitously, a small number of worms from hamster livers from all three groups exited the biliary tract following euthanasia of the hamsters, resection of the liver at necropsy, and during preparation of the livers for immunohistopathological analysis after fixation, microtome sectioning, and staining. We specifically and assiduously searched for and recovered these individual worms, at the same time taking due care not to damage the liver tissues destined for the immunohistopathological analysis. Subsequently, indel levels in the targeted genes were established using this sample of the Experiment 2 worms ("escapees"…)..

33. The differences in pathology reported in the subsequent results sections (fibrosis and collagen) could be confounded by different numbers of adult worms between the control group and knockdown; this should be controlled for in a regression model".

The authors concur that the variation in worm burden complicated the analysis of disease burden. Specifically, we concur that worm burden may have influenced collagen levels. Accordingly, to mitigate the impact of differences in worm burdens, we performed targeted collagen assessment in bile ducts where flukes were situated (Figure 6C). Unfortunately, we do not have precise data on the adult worm burden, but we have conducted the fibrosis vs EPG comparison as a proxy for worm burden (Figure S6). Substantial patterns were inapparent in the control or Δ*Ov-grn-1* groups. The Δ*Ov-tsp-2* group exhibited a significant negative correlation between fibrosis and EPG; investigation of the basis of the surprising result is beyond the scope of this report.
